# Adolescent Alcoholism and Drug Addiction: The Experience of Parents

**DOI:** 10.3390/bs5040461

**Published:** 2015-10-29

**Authors:** Peter W. Choate

**Affiliations:** Department of Child Studies and Social Work, Mount Royal University, 4825 Mount Royal Gate SW., Calgary, AB T3E 6K6, Canada; E-Mail: pchoate@mtroyal.ca; Tel.: +1-403-440-5008; Fax: +1-403-266-0214

**Keywords:** addiction, adolescent substance abuse, addiction and family, parenting troubled youth

## Abstract

Alcoholism and drug addiction have marked impacts on the ability of families to function. Much of the literature has been focused on adult members of a family who present with substance dependency. There is limited research into the effects of adolescent substance dependence on parenting and family functioning; little attention has been paid to the parents’ experience. This qualitative study looks at the parental perspective as they attempted to adapt and cope with substance dependency in their teenage children. The research looks into family life and adds to family functioning knowledge when the identified client is a youth as opposed to an adult family member. Thirty-one adult caregivers of 21 teenagers were interviewed, resulting in eight significant themes: (1) finding out about the substance dependence problem; (2) experiences as the problems escalated; (3) looking for explanations other than substance dependence; (4) connecting to the parent’s own history; (5) trying to cope; (6) challenges of getting help; (7) impact on siblings; and (8) choosing long-term rehabilitation. Implications of this research for clinical practice are discussed.

## 1. Introduction

Addiction affects family functioning. It changes how families relate and the roles that each member plays. The addiction becomes the family’s priority [1,2]. Gruber and Taylor [3] present a cogent argument that addiction must be seen from a family perspective to be properly understood. Much of the research and clinical literature has been focused on adults who have substance abuse or dependency disorders [4,5,6]. Insights on family functioning with an addict can be seen through personal stories that tell the experience from one family’s or one teenager’s perspective [7,8,9,10]. Velleman [11] has identified seven areas of family functioning that are impacted by addiction: roles, rituals, routines, finances, communications, conflict and social life.

Usher, Jackson and O’Brien [12,13] have specifically looked at families who have an adolescent abusing alcohol or drugs. In their work, these authors note that the negative effects of drug use that a parent might experience are seen across a diverse range of the youth’s life—schooling, health and family relationships—but the causes of the effects are not immediately seen. They state that “…contact with the legal system or family problems are commonly the triggers for recognizing substance misuse…” (p. 210). Jackson and Mannix [14] note that the problems are typically quite entrenched before they become recognized. The onus for managing the problems will fall largely to the parents.

In a qualitative study, Jackson, Usher and O’Brien [15] reported that families are fractured by adolescent substance use when the adolescent has “serious and on-going illicit drug use.” (p. 323). They add that substance use touches all aspects of family life. This includes parents who are feeling that the family is being torn apart while also experiencing the youth as “complex, demanding, overwhelming and highly stressful” (p. 323). This same research speaks of parents who are experiencing betrayal and loss of trust in the child. Families experience “ongoing turbulence” (p.329). Parents describe being “torn between wanting to provide support for their drug affected child and needing to ensure a stable environment for their other children whose peaceful use of the family home was affected” (p. 329).

Orford and his colleagues [16] identified that families reached a point where they will engage the problem of addiction directly, tolerate it or withdraw from the problem. In a review of the literature, Smith and Estefan [17] described that addiction impacted families very broadly but that there were barriers to disclosing or talking about the problems. Divulging the family secrets was seen as harmful, which reinforced the need to protect the secrets. They also felt that the mother carried the heavier burden, as there is a lot of social pressure to be successful in the role of primary caregiver.

Usher, Jackson and O’Brien [13] identified eight major themes in how families experienced serious substance abuse in a youth: (1) the process of confirming suspicions; (2) struggling to set limits; (3) dealing with consequences of the drug use on the family; (4) living with blame and shame; (5) trying to keep the child safe; (6) grieving the loss of the child that was; (7) living with guilt; and (8) choosing self-preservation. Barnard found similar wide-ranging impacts [18].

Butler and Baud [19] reported that parents found themselves in conflict over how to react and manage the behaviors arising from the substance dependence. Communication between parents was damaged which made problem-solving more challenging as the youths’ behaviors grew beyond their management [20,21,22].

Substance dependency in youths is different from the adult experience [23]. For example, the adolescent has a harder time connecting present actions with longer-term consequences; beliefs and attitudes reflect their developmental stage; and their physiological responses are reflective of physical development. Concurrent mental health disorders often emerge in the adolescent stage of development, which may complicate the parents’ understanding of the issues.

Research conducted with families where the substance-dependent person is an adult clearly shows families are significantly impacted. The research is quite limited when it is a youth who has the substance dependency. The present work seeks to understand how parents have experienced and coped with substance dependence issues emerging with their youth.

## 2. Method

### 2.1. Theory

This qualitative study uses Grounded Theory, which tries to understand what is happening in the lives of those who are living it, or at least the portion of the life being researched [24]. Probing questions allow participants to reflect on their experience while giving room to tell their story with their own language, opening the daily-lived experience [25]. Through it, the researcher begins to know the process and the phenomenon, rather than just the setting in which the experience occurs [26].

### 2.2. Recruiting

Participants were recruited from a long-term, family-based adolescent rehabilitation program. There were 31 parents or caregivers of 21 adolescents interviewed. All of the adolescents had been assessed by the program as meeting the criteria for a substance dependence disorder in accordance with criteria outlined in the DSM IV criteria [27].

This work involved a convenience sample of parents whose adolescents were participating in the same long-term rehabilitation program. It should be noted that parents of truly substance-dependent adolescents are a difficult population to reach. The author is a clinical consultant at the center, meaning that the parents as well as the adolescents already knew him. As Neale, Allen and Coombs [28] note, there is a challenge in building a trusting relationship with substance-dependent populations. The author’s connection to the center helped to overcome that resistance. None of the participants had an ongoing therapeutic relationship with the author.

All parents signed an informed consent and were free to withdraw from the study at any time. None did so. Parents were assured of confidentiality and, thus, in reporting direct comments, identifying data has been removed. This has meant that there are minor changes in some direct quotes. An example is when a sibling's name was removed; it was replaced with “sister” or “brother”. Quotes have also been edited for readability. When parents were interviewed together their participant numbers were the same but with “a” or “b” afterwards to indicate their own contributions.

Participation was voluntary and was not related to any requirements of the treatment center. Four caregivers in the program chose to not participate. The study was conducted in accordance with the Declaration of Helsinki, and the Ethics Committee of the treatment center approved the protocol. The treatment center was not aware of which parents chose to participate.

### 2.3. Data Gathering and Analysis

Interviews were recorded and lasted between 45 min and 2 h. The interviews followed a semi-structured approach [29]. The areas probed included how the caregiver became aware of the drug and/or alcohol use; how they came to understand that the problem had escalated to the point that it might be dependency; what interventions the caregivers tried; what the impacts were on personal and family functioning; and what led the caregiver to determine that long-term, family-based rehabilitation was needed. Saturation occurred around the 20th interview [26,30]. Transcribed interviews were analyzed using NVivo qualitative software.

**Table 1:** Caregiver participants and their relationship to the youth in treatment.

Diagnosis (*n* = 17)	
Biological Mother	17
Biological Father	7
Step Mother	1
Step Father	1
Adoptive Mother	2
Adoptive Father	1
Other Caregivers	2

The sample consisted of 31 caregivers (referred to as parents for convenience), as seen Table 1. The 21 youths self-reported that, on average, they commenced use at 13.38 years. Eight had prior involvement with the criminal justice system. Eighteen of the 21 youths stated that they were involved in a variety of crimes for which they were not caught. These included dealing drugs, breaking and entering, assault, as well as robbery and shoplifting. Much of this activity was self-reported to be in support of their substance use. Four of the youth had involvement with the child protection system prior to entering the program. The sample was racially homogeneous, all being Caucasian save for one youth.

The youths had a variety of diagnostic impressions at the time of admission. Seventeen of the 21 youths had a form of mental health diagnosis as seen in Table 2. In addition, five youths had a history of self-harm. One youth had been diagnosed with Fetal Alcohol Spectrum Disorder (FASD). Three had been victims of neglect in childhood and three of physical abuse, which was confirmed by the caregivers being interviewed. The perpetrators were not necessarily the present caregivers.

**Table 2:** Frequency of comorbid diagnosis of youth in treatment.

Diagnosis (*n* = 17)	
Attention Deficit Hyperactivity Disorder	9
Conduct Disorder	2
Oppositional Defiant Disorder	6
Mood Disorders	8
Anxiety Disorder	1
Learning Disability	4
Bulimia Nervosa	1

Five fathers and seven mothers reported their own history of substance abuse (although not dependence). Four mothers and one father reported having a history of being diagnosed with a mental health disorder. Eighteen of the 21 families reported histories of substance abuse issues (again not necessarily substance dependence) in the extended family. Ten families indicated diagnosed mental health disorders in the extended family.

## 3. Results

### 3.1. Theme One—Finding Out about the Problem

Parents described that finding out about their youth’s problem was a process that emerged in layers, in a non-linear fashion. Discovery of substance use tended to follow three typical routes. The first was being faced with direct evidence that there was a significant problem. This could be an overdose requiring emergency hospitalization or an arrest related to substance use or allied behaviors such as assault while intoxicated. The latter was the least frequent method of discovery but it was one that tended to move parents into action quickly. An example is seen with a mother who became quite active in monitoring her daughter and seeking help when, in grade eight, the youth was hospitalized for alcohol poisoning *(Participant 23*, *Mother)*.

Parents who experienced these clear indications of a significant substance abuse problem changed their view of the youth quickly. They became quite vigilant while trying to gain some control.

The second route of accidental discovery was quite common. Parents would discover paraphernalia or small amounts of drugs or bottles of alcohol. Another accidental route was when one of the youth’s siblings or friends made a comment that suggested use. Parents were typically baffled, as this was unexpected information, but not sufficient enough to allow them to determine the seriousness of the problem. However, the latter was not a consistent or predominant pattern of responses identified. Some parents would confront the youth, some would wait to see if more information emerged, while others set out to investigate the youth’s involvement in substance use.

The third route saw parents confronted with a variety of changes in their youth that they tried to make sense of. These included changes in marks at school or problems with attendance. Other changes included diminished interest in a previously enjoyed extracurricular activity, increased secretiveness, less willingness to be involved with the family and increased defiance. Parents were prone to seeing these changes as part of the normal teenage years. One of the fathers described his reaction to such a discovery:

“Every kid smokes pot a little bit. You know, I kind of done the pot especially to extremes. I just thought he’d use it, not like it very long and move on with his life because he was a very good student.”

(*Participant 7*, *Father*)

Parents who learned about the problems through the latter two routes found themselves in a quandary as to how to respond. Many confiscated what they found, or confronted the youth or did both. Parents typically made it up as they went along. Responses tended to be more reactive than planned. Emotions took control, often leading to unsuccessful interventions. One mother stated, “I found this stuff and just hesitated. I didn’t know what to do. So I grabbed it. I hid it in my room” *(Participant 4a*, *Mother).*

Several parents described becoming authoritarian after these first discoveries. This was an attempt to control the situation. However, many remained confused for quite some time as the youth’s behaviors waxed and waned from problematic to quite pleasant. One mother, for example, described that her youth ranged from compliant and “sweet” to suddenly being “raging and demanding” (*Participant 23*, *Mother*)*.*

### 3.2. Theme Two—Experiences as the Problem Escalated

All of the parents reported that youths’ peer connections changed. Those who had been important in the youth’s life prior to drug use began to disappear and new influencers emerged, although many parents talked about not knowing these new people. They were kept at a distance: “So I never see her friends. If I ask her, she wouldn’t tell me. So she was keeping her life to herself” (*Participant 22*, *Mother).*

Most parents reported that they stopped seeing the child that they had known before the substance use. At the same time, as things got worse, confrontation became part of family life. When asked how her daughter had been treating her, one mother noted, “Like I was the cause of all her problems, she couldn’t deal with me, couldn’t deal with life at home” *(Participant 24*, *Mother).*

Volatility, screaming and yelling became the norm of the relationships between all but a few parents and their youth. As one mother put it:

“He never got physically angry with me but he very often got like really screaming at me and then he would get so, you could see the rage in him and he would just get up and slam something and walk …”

*(Participant 17*, *Mother)*

This “new” person was more challenging to manage. Again, they searched for ways to respond to the behaviors. The co-parenting relationship was often fracturing at the same time. All of these parents described a diminished influence on their youth. Their influence was replaced by new peers and families that supported drug use. This isolated the majority of parents. The possibility of an alliance with another parent who might support getting control of the substance abuse was unrealized. This mother illustrates the point when she speaks of her daughter running away and the mother at the place she had gone to denying that she was there: “It was like a real shock. I just can’t imagine lying to another parent like that about their kids.” This same mother experienced further barriers to working with other parents when she discovered that “she had another friend whose parent supplied the drugs and alcohol to them so that seems like, to us, the most incredibly stupid thing a parent could ever do” (*Participant 6, Step Mother)*. For this mother, it meant that not only was she discovering that her daughter had problems with substance use, but that there were families with values very different from their own which supported the substance use.

### 3.3. Theme Three—Looking for Other Explanations beyond Substance Abuse

Most parents spoke about the stigma surrounding substance abuse and dependence. Thus, when presented with the possibility of explanations for the behaviors that did not carry that stigma, parents sought them out. Typically this involved mental health disorders. This is illustrated by a mother who said, “I had come to the conclusion that his problems were more psychological”. This meant “I wasn’t thinking addiction with him” (*Participant 3*, *Mother*).

Other explanations offered parents a sense of hope, believing that, if this other issue could be addressed, then the drug use would diminish or disappear. This could be particularly powerful if the issue related to something that the parent might feel guilty about, as seen with this mother:

“I kept thinking he was just really depressed and that he was just really upset about our separation and divorce and having to go back and forth between homes and that if he could just talk about those things and get those feelings out, then things would be ok.”

(*Participant 17*, *Mother*)

Professionals were often described as supporting the view that the problems were based in mental health concerns, which could make it hard to shift the focus. One mother noted that the psychiatrist would explain the mental health diagnosis to the youth but “didn’t address the drug use at all, even though we kept saying 'he’s using drugs'" (*Participant 4a*, *Mother*)*.*

There were a few parents who saw the new behaviors as an expression of a concern they had been facing for many years, such as behavioral issues, academic problems or, in one case, FASD. What they did not understand was why the concerns were getting worse. They did not know about the substance use or the extent of it. As a result, they did not make the connection. Over the years, their concerns about the youth had been narrowed to focus on the predominant behavioral issues. Thus, they tended to view current behaviors from that perspective, as illustrated by this mother who noted, “She’s always been very volatile. From the time she was little, she’s been very stubborn, very strong willed” (*Participant 24*, *Mother*)*.*

The more parents became focused on the historical concerns as a basis for understanding the current situation, the longer it appeared to take them to get focused on substance use concerns.

### 3.4. Theme Four—Connecting to the Parent’s Own History

Parents saw the issues of their youth through their own past experiences. Those who had a significant history of substance dependence were strongly influenced by those experiences. One father, who had used for many years, illustrated how his experience was the way he made sense of his son’s behaviors:

“I smoked pot for 30 years and I couldn’t really cut it down. You know, I was going to do it. I was gonna get up in the morning and do it all day long until I went to bed. And I saw my son doing the same thing, but I saw him like just going down the toilet.”

(*Participant 7a*, *Father*)

The mother of this youth did the same thing, although this illustrates how different experiences in the family system resulted in a different interpretation:

“And his dad did the same thing. His dad looked at him and said, ‘That’s me.’ But his dad is a very different person. He was able to be high and get a Master’s Degree. He was one of those addicts. And what I thought was that our son was more like me and my side of the family who are reckless. We have a reckless energy and personality. Like the alcoholics on my side of the family are dead already and have killed people. On my father’s side, wild drunks, but when I looked at my son I could see that he had that. He didn’t have his father’s way of using. He had the other way of using which was the real reckless.” (*Participant 7b*, *Mother*)

Given that 12 of the 31 parents had a history of substance abuse problems and that there was a history in 18 of the 21 families, these experiences acted as a common way to try to understand the issues, while those parents without such a history reported being confused and struggled to understand. A few were able to draw upon what they saw as similar experiences. One mother spoke of having mental health, medical and disability issues, which she had needed support to manage. This encouraged her to seek help for her youth. After she received help, she observed, “But man, it was tough. It was confusing for me” (*Participant 13*, *Mother*)*.* Another spoke of having been raped and keeping it secret. This led to what she described as “wrong decisions”. That history “made it easier for me to get him in there and get the help he needed earlier rather than later” (*Participant 20*, *Mother*).

### 3.5. Theme Five—Trying to Cope

Parents saw their lives beginning to fall into a pattern of chaos. The whole of the family system was impacted. Their youth’s behaviors became higher risk and family connections grew steadily weaker. As these changes occurred, parents felt more out of control. They would reach out for help to other family members, to professionals, or both. Parents reported a growing desperation and an increased inability to effectively cope, but they found that reaching out for help could be less than useful at times, adding to the pressures to cope.

Most parents spoke about the increased stress that made it harder to manage. Simultaneously, the majority reported a weakening of the co-parenting relationship. Anger crept into relationships, which negatively affected family functioning. One father describes that, as a result of his anger, “I felt ganged from my daughters” (*Participant 11*, *Father*).

Several parents spoke of coping by withdrawing and seeking ways to not be home and have to deal with the chaos. For the other parent, this meant shouldering a heavier parenting burden, which they resented. No matter how much they tried to manage, they were not coping and the strain continued to increase. One mother summed this up by saying, “I was a mess. I was terrible and then I’d try to call my husband and tell him what’s going on and he’s trying to work and be away for a week and I felt like a failure” (*Participant 23*, *Mother*).

Parents spoke of using strategies that, in retrospect, they saw as “crazy”. For example, a father spoke of confiscating his son’s drugs but then went on to say, “If you have any obligations for what I’ve confiscated, I’ll cover it” (*Participant 9*, *Father*)*.* A mother spoke of putting $800 in cash into an envelope so her son could pay off his drug debts that he then headed off to do, wondering “if I would ever see him again” (*Participant 21, Mother*).

Other parents tried to cope by being more and more in control by reading diaries, Facebook, phone messages and hunting down their youth in the hope of saving them, often at significant risk to the parent.

### 3.6. Theme Six—The Challenges of Getting Help

Parents typically reached out for formal help when they felt that the pressures arising from their youth’s behaviors were now far beyond their abilities and capacities to cope. Most parents described feeling powerless and that nothing they were trying seemed effective. When they crossed that barrier, reaching out made sense and parents often spoke about trying almost anything to find a solution. Most parents tried to get some formal assistance for their youth through public health agencies or private resources. As this father shows, once he realized that his tolerance for the chaos had been exceeded, he had to act and started calling “the police; I called social services. I was just trying to get help because we realized things had got out of control very quickly” *(Participant 13, Father).*

Reaching out for help was a major step, and one that parents often described as disappointing and which failed to make a difference. In various ways, parents often felt that the professionals and agencies did not understand substance dependency. They also felt professionals were exceedingly reluctant to use terms like dependency and addiction.

Professionals offered solutions, which were at odds with family values or needs. As one father notes, his family was already traumatized through other events, but the professionals suggested that they throw the teen out of their home, which may have caused more damage. Too often parents did not feel heard or understood as they tried to deal with the issues. Parents were frustrated that the professionals frequently held back information about their youth’s situation due to issues of confidentiality. Given the age of the youths, therapists were hampered when the youth would not give permission for information to be shared with parents. Many parents spoke about how this disempowered them and even feeling that the therapist had become allied with the youth. Many described the confidentiality barriers as enabling secrets to be held, thus making it harder to understand what the youth’s issues were and how to help. Most parents spoke about wanting information that would allow them to make sense of what was happening and how they could effectively respond. One father noted that it would have made a significant difference “if someone sat down and counseled us and talked to me face-to-face, like you have a sick kid and the sickness that they have is addiction. They’re a drug addict, you know” (*Participant 13*, *Father*).

Even professionals who did feel that youths might have dependency problems seemed reluctant to actually say it to parents but, nonetheless, offered a suggested direction. For example, a mother spoke of a therapist who directed her to the program where this research was conducted: “It was, his therapist, who suggested I ‘look into’ this program”. She added that the therapist did not really come out and say why. She said it would have been helpful if the therapist had just laid it out and told her that her youth might be substance dependent (*Participant 10*, *Mother*).

Parents also noted that their ability to get their youth connected to help was challenging, as the youth often just refused to go. Even though some of the youths went to counseling to appease the parents, there was little constructive change. As one parent noted, her daughter told her one day, “You’re just wasting your money because I just sit there and talk to her and I don’t really care what she says” (*Participant 6*, *Mother*). There were a few parents who decided to get their own therapeutic support that allowed them to both validate the challenges and get coaching on various ways to respond to behaviors.

One resource that was seen as helpful was police officers because they tended to tell the parents bluntly that the issue might be substance abuse. This may be a result of police officers having a different, non-therapeutic role and, thus, a different social position with both youths and their families. The police became involved in a variety of ways, ranging from picking the youths up for public intoxication to arrests.

Parents also found help through their own peers who had children with serious substance abuse or dependency problems. They spoke openly and frankly about their own experiences and offered insights that validated what the parents had been going through. Connecting to these peers happened through informal meetings, attending self-help programs or by calling addiction treatment centers that offered connections to parents of former clients.

Parents were often ambivalent about reaching out to family for support. Some felt it could be useful, while many did not wish to do so. Those who did not spoke about not wanting to burden their family, while others described guilt and shame regarding the problems with their youth. They worried that they would be judged for failing in the parental role. When some did reach out to family, it was not always helpful. It could be met with family members becoming enmeshed and enabling the youth. This aunt illustrates this:

“He would go out and use and then he would phone grandma at 3 o’clock in the morning and say, “Grandma, you gotta come get me.” And grandpa would say, “No, don’t go get him.” And grandma would say, “I gotta go get him because if he dies in a snow bank, I’m responsible”.

(*Participant 18*, *Aunt*)

### 3.7. Theme Seven—Impact on Siblings

Parents frequently spoke about the impact of the substance use on youths’ siblings. These could be direct effects, such as being stolen from or assaulted. Indirect effects included the sibling’s needs being neglected as the parents shifted their focus more and more to the youth with the problem, and leaving siblings to fend for themselves. In some ways, the siblings lost their brother or sister as well as the family as a unit. This father described noting the loss for his daughter: “I think it bothered her a lot to see him destroyed like this and she lost her best friend” (*Participant 9*, *Father*).

The sibling could also become integrated into his or her own pattern of enabling. A father described that his daughter “was keeping a lot of secrets for him, and, once in a while she would let a secret out, then she’d be terrified that he’d hurt her.” He went on to describe how his daughter would “have a lot of guilt over telling anything on him” (*Participant 4b, Father*). This illustrates how siblings can be very conflicted in their relationship with the using sibling but also other family members. However, the data did not suggest that siblings had a homogenous response pattern.

Other siblings would withdraw and find more connections external to the family, as seen with this mother’s observation: “Our oldest would kind of disassociate herself by doing a lot of activities and having a lot of friendships and pouring herself into the things that she was doing” (*Participant 11*, *Mother*). Others spoke about siblings refusing to be involved with the using sibling and others who took on parenting roles in an attempt to gain some control.

Siblings could also put parents in untenable positions of feeling like they could not meet the needs of both: “She asked me, “Mom, kick my sister out.” I said, “I cannot do that. She’s my daughter”” (*Participant 22*, *Mother*).

### 3.8. Theme Eight—Choosing Long-Term Rehabilitation

All of these parents forced the issue of entering long-term rehabilitation. It represents the point where parents were no longer able to tolerate chaos and needed to find a solution that might work. Parents indicated that all they had tried up to that point had failed and something needed to be different. “I just didn’t want to live like that anymore” (*Participant 3*, *Mother*).

Other parents felt that they had reached a crisis point where something needed to be done urgently. This is poignantly seen in this father’s observation where he says he acted because “I knew my son was going to die” (*Participant 2*, *Father*). The fear of death was a common concern.

For many, they just felt that the behaviors were never going to get better and were often getting worse. One father commented, “I caught him as a dealer” (*Participant*, *9 Father*). The decision could also be driven by the realization that there was nothing left to try. The family was exhausted and they had done all that they could think of to address the problems. Most spoke of feeling like they were out of options.

## 4. Discussion

This work confirms many of the themes seen in prior research [12,13,14,15]. These include themes of substance abuse and dependency significantly impacting family functioning; problems arising within the various systems in which the youths are involved including school, peers and extracurricular activities [5,6,31,32]. Like other work, this research saw changes in the substance-dependent person that brought new influences into the family, such as the police and other peers and families with substance dependency concerns [1,12,13,14,15]. This present work shows how parents’ ability to effectively fulfill their role became increasingly compromised as substance abuse grew towards dependency. The current work extends prior research into the effects of trying to parent an adolescent with a full-blown substance dependence.

This work also shows common ground between families where the parent or another adult in the home has the substance dependency and families where the adolescent is the one with the issue [1,18]. Prior work has shown that conflict in the family grows as the substance dependency grows [33], which was unquestionably the case in this research. Indeed, the conflict grew to the point where family cohesion was damaged, be it other parent-child relationships, sibling relationships or co-parenting. Family relationships were strained across the family system, including outward to the extended family [1].

At the parental level, coping became about survival as opposed to leading the life that the parent envisioned. They were trying to find their way through the chaos, but as they sought meaning to what was going on around them, the confusion and chaos that is simply a part of living with substance dependency prevented a clear understanding. For the substance-dependent youth, “using” was what mattered and, thus, efforts to sustain family relationships fell by the wayside. It is a feature of this research that families underwent fundamental, unmanageable changes as a result of these youths’ substance dependency.

Other behaviors found in this research that are consistent with the literature includes enabling, withdrawing, minimizing, denying, being victimized, neglecting family relationships, and being engaged in destructive behaviors both to the self and other family members. Teenagers are considered to be a developmentally vulnerable population and this pattern is contextually different from when substance dependency is found with an adult [23].

A dominant theme in this research was that parents felt unsupported. The therapists involved with their youths were typically constrained by confidentiality laws and rules from telling parents very much. This left parents quite frustrated and disempowered, with many believing that an alliance had developed between therapist and youth. They saw this as running counter to the interests of the parents.

This tended to emphasize a feeling of being ineffective in the parenting role. This meant that they were often neglecting the needs of the other children in the family. This neglect would cause the siblings to look elsewhere for guidance or to isolate and, in many cases, detach from the parental guidance [33,34]. Siblings were at risk of starting to use as they adapted to the substance abuse environment [35]. Bamberg, Toumbourou and Marks [36] indicate that siblings of substance-dependent youth are at greater risk for their own adverse outcomes. This is an area that warrants further inquiry, as the research on the effects on siblings is limited.

Throughout these interviews, the very real emotional pain these parents experienced was palpable. Thus, it may not be all that surprising that parents sought explanations for this pain in a way that was not as burdensome as substance dependence and was more socially acceptable. This makes sense given that the parents have such a large stake in the youth. They raised the youth and invested much of their lives in this person. They are burdened with the societal expectation that they will be successful at this job [17]. Thus, it might well be expected that they would look for something other than substance dependency. Clinically, it becomes important for a parent to receive support in realistically assessing the cues. They might then be led to a different understanding of what is going on and activate earlier intervention tasks. However, parents need very specific supports to address their own emotions as the story of substance dependency unfolds. It is the unwanted story that demands understanding while creating a profound sense of failure as a parent. 

The process of coming to understand the substance dependency was neither linear nor transparent for these parents. On the contrary, they found themselves sorting through contradictory information, which lead them in diverse directions. For most, why the youth’s behaviors were changing was not clear. Even those with their own history of substance dependency found responding to these problems presented them with unfamiliar challenges about which they were uncertain how to respond. These parents could see problems emerging but they did not know what to make of them. As Barnard [1] saw, the picture is confusing and fraught with a parent’s natural desire to see their youth succeed. Yet these parents were not blind to what was going on. They simply did not understand it. There were exceptions that were typically related to behaviors that made the issues of substance abuse evident. The majority of parents learned in layers, trying to make sense of what was happening, while also trying to manage a growing sense of chaos for themselves and their family.

Parents typically did not avoid but responded to what they “thought” was occurring. Try as they might, things just got worse. It was happening before their very eyes no matter what they did. The power of the substances and the related culture was greater than the interventions the parents could bring to bear. None of these interventions, be it outpatient counseling, school interventions, family therapy, lower intensity rehabilitation, changes in living situations or various combinations of these efforts, were effective in these cases, although they may well be in cases where substance abuse is more of a concern than dependency. Parents need resources that are focused on these more severe cases.

Orford *et al.* [16] speak about families reaching a point where they will tolerate, engage or withdraw. This seems to have also been the case with these families. They moved from one position to the other. Tolerance appeared to be most evident with those parents who saw the behaviors as reflective of some aspect of normative adolescent development. Withdrawal occurred as the chaos grew and one parent or the other would seek refuge outside of the house. However, for these parents, the challenges grew to the point where they would almost be forced to engage either because the chaos was too great or external forces such as the presence of police officers, problems at school or health care crises forced action.

Too often, parents sought services from the community that failed to be of assistance. Parents spoke about being shut out of efforts, such as counseling, or of the substance dependency problems. Often the issues were minimized to the parents. There is no doubt that parents rarely met with professionals who were prepared to directly address the problems of substance abuse or dependency. Even when professionals appeared to feel that the problems were very significant, the language used appears to have been couched in softer terms. Parents did not feel that was helpful. The clinical implications are that the professional should be clear in the messages. However, most parents did not appear to understand how professionals working with adolescent clients are often bound by legal and ethical limitations precluding disclosure without their client’s approval. A few sought out their own therapist to help coach them. When that happened it was useful. Overall, however, this group of parents with youths dependent on rather than abusing substances did not feel that the various interventions they sought were effective. 

The research also shows that the family needs repair on a systematic level. Effective intervention was shown to be required for all family members. This echoes the work of Cook [37], who concludes that treating an adolescent with substance dependency without treating the family “limits our vision and decreases the potential for the recovery of a young life” (p. 156). Given all the impacts reported by these parents on every family member, such a clinical plan is strongly required.

There are a number of clinical implications to this research that include finding ways to be more inclusive of family members when working with youths showing serious substance abuse or dependency problems. Equally, parents may benefit from their own supports allowing them to receive coaching as well as to better understand the cascade of emotions that they deal with while trying to cope. When professionals work with families, this research suggests that they may need to be quite clear in their language so that all family members involved hear, in unequivocal terms, the concerns. As well, there is a clear need to bring siblings into the process as they appear to have their own sets of emotional needs.

### Limitations and Areas for Future Research

This research used a convenience sample that relies upon retrospective self-report which opens up the data to the influence of conscious and unconscious biases. Furthermore, it does not compare this set of parents with those who have chosen to use different treatment approaches. Rather, it provides a platform for understanding the process of learning about the lived experiences of one group of parents who have chosen to use a unique, intensive treatment facility. It may well be that there are situations where less intense interventions have been successful with a similar population. It also means that the results may not be generalizable. Even though other interventions have not been explored, it may be helpful for future research to do this.

In addition, from these parent interviews, there are indications of how siblings have been affected by the behaviors of their brother or sister’s substance dependency and related behaviors, but the siblings have not been interviewed directly. This appears to be an interesting area for further exploration.

These interviews also suggest that further research could begin to explore the pre-substance-using period to better understand what leads youths into the trajectory towards dependence. We might ask what is different about this group that leads them to go that way. Yet another area for exploration is whether the lived experiences of families may vary depending upon which substance becomes the preferred choice.

Given the views of these parents that many of the services that became involved with their youth were not effective, and that they felt professionals they saw did not seem to understand substance dependency in youth, it would be valuable to see how professionals who work with this population see the issues.

## 5. Conclusions

This research extends the work on addiction in the family. It shows that, while there are many experiences that are similar to those when the substance dependence person is an adult, there are also unique issues when that person is a youth. Parents need support to be able to see the emerging substance dependence with their youth and how they might effectively respond. They also need support in helping the other children in the family manage. This work indicates clinicians should be aware of the need to intervene not only with the identified client but also create interventions for the entire family system.
